# Signaling Pathways for Long-Term Memory Formation in the Cricket

**DOI:** 10.3389/fpsyg.2018.01014

**Published:** 2018-06-22

**Authors:** Yukihisa Matsumoto, Chihiro S. Matsumoto, Makoto Mizunami

**Affiliations:** ^1^College of Liberal Arts and Sciences, Tokyo Medical and Dental University, Ichikawa, Japan; ^2^Graduate School of Life Sciences, Hokkaido University, Sapporo, Japan

**Keywords:** long-term memory, NO-cGMP signaling, cAMP signaling, crickets, classical conditioning

## Abstract

Unraveling the molecular mechanisms underlying memory formation in insects and a comparison with those of mammals will contribute to a further understanding of the evolution of higher-brain functions. As it is for mammals, insect memory can be divided into at least two distinct phases: protein-independent short-term memory and protein-dependent long-term memory (LTM). We have been investigating the signaling pathway of LTM formation by behavioral-pharmacological experiments using the cricket *Gryllus bimaculatus*, whose olfactory learning and memory abilities are among the highest in insect species. Our studies revealed that the NO-cGMP signaling pathway, CaMKII and PKA play crucial roles in LTM formation in crickets. These LTM formation signaling pathways in crickets share a number of attributes with those of mammals, and thus we conclude that insects, with relatively simple brain structures and neural circuitry, will also be beneficial in exploratory experiments to predict the molecular mechanisms underlying memory formation in mammals.

## Introduction

Brain structures and neural circuitry of insects are relatively simple, and they are therefore useful for exploratory experiments to predict the molecular mechanisms underlying memory formation in mammals. Memory in insects as well as that in vertebrates is a dynamic process organized in two main types: short-term memory (STM) and long-term memory (LTM). The former is defined as protein synthesis-independent memory, and the latter is defined as protein synthesis-dependent memory. They can be distinguished by their temporal courses and molecular mechanisms (Kandel, 2001). It is a common understanding that while STM is based on temporal changes in the synaptic strength due to covalent modifications of pre-existing proteins, LTM is supported by long-lasting alteration in the strength of synaptic function demanding for transcription and translation of genes, among a wide variety of animals including mice, sea hares *Aplysia* and fruit flies *Drosophila* (Montarolo et al., 1986; DeZazzo and Tully, 1995). The cAMP pathway is demonstrated to be critical for LTM formation in all of these animals (Bartsch et al., 1995; Yin et al., 1995; Abel et al., 1997). The cAMP pathway is a signaling cascade beginning with an increase in intracellular cAMP that activates cAMP dependent protein kinase (PKA). PKA phosphorylates the transcription factor cAMP-responsive element-binding protein (CREB) that leads to LTM formation. The nitric oxide (NO)-cGMP pathway is another system playing critical roles in the formation of LTM in sheep (Kendrick et al., 1997), great pond snails *Lymnaea* (Kemenes et al., 2002), and honey bees (Müller, 1996, 2000).

In this review, we will summarize the results of our pharmacological behavioral studies on the molecular mechanisms of the formation of LTM in the cricket *Gryllus bimaculatu*s and propose an updated model of LTM formation. The main results introduced in this review are shown in **Table 1**.

**Table 1:** Summary of the effects of inhibitors on 30-min and 24-h retention.

Inhibitor	L-NAME	ODQ	L-DIL	W-7	KN-62	DDA	KT5720	CHX
Target	NOS	sCG	CNG channel	CaM	CaMKII	AC	PKA	Protein synthesis
Effects on 30-min retention after multiple-trial conditioning	No effect	No effect	No effect	No effect	No effect	No effect	No effect	No effect
Effects on 24-h retention after multiple-trial conditioning	Fully impaired	Fully impaired	Fully impaired	Fully impaired	Fully impaired	Fully impaired	Fully impaired	Fully impaired
Effects on 24-h retention after single-trial conditioning								
+NO-donor	No effect	Fully impaired	–	–	–	–	Fully impaired	Fully impaired
+cGMP analog	No effect	No effect	Fully impaired	Fully impaired	Fully impaired	Fully impaired	Fully impaired	Fully impaired
+Ca^2+^ ionophore	–	No effect	No effect	Fully impaired	Fully impaired	Fully impaired	–	–
+AC activator	–	No effect	No effect	No effect	Fully impaired	Fully impaired	–	–
+cAMP analog	No effect	No effect	No effect	No effect	No effect	No effect	Fully impaired	Fully impaired


*NOS, NO synthase; sGC, soluble guanylyl cyclase; CNG channel, cyclic nucleotide-gated channel; CaM, calmodulin; AC, adenylyl cyclase; PKA, protein kinase A; L-DIL, L*-cis*-diltiazem; DDA, 2′5′-dideoxyadenosine; CHX, cycloheximide. Data for L-NAME, L-DIL, and W-7 experiments are from Matsumoto et al. (2006); Data for ODQ, DDA and KT5720 experiments are from Matsumoto et al. (2006, 2009); Data for KN-62 experiments are from Mizunami et al. (2014); Data for CHX experiments are from Matsumoto et al. (2003, 2006). The concentrations of the administrated drugs were as follows: L-NAME (400 μM), ODQ (200 μM), L-DIL (1 mM), W-7 (200 μM), KN-62 (2 mM), DDA (1 mM), KT 5720 (200 μM), CHX (10 mM), NO-donor SNAP (200 μM), cGMP analog 8-br-cGMP (200 μM), Ca^2+^ ionophore A23178 (200 μM), AC activator forskolin (200 μM), cAMP analog 8-br-cAMP (200 μM), and DB-cAMP (200 μM).*

Crickets provide several advantages to investigate memory-related molecules. First, they demonstrate remarkable ability of olfactory learning and memory, including that requires cognitive functions. For example, they exhibit robust olfactory memory maintained throughout their lifetime (Matsumoto and Mizunami, 2002a), contextual learning (Matsumoto and Mizunami, 2004), high capacity of memory storage (Matsumoto and Mizunami, 2006), second-order conditioning (Mizunami et al., 2009), and sensory preconditioning (Matsumoto et al., 2013a). In addition, they have remarkable visual learning ability (Unoki et al., 2006; Nakatani et al., 2009; Matsumoto et al., 2013b). Second, effective approaches that greatly facilitate analysis of the molecular basis of learning and memory are feasible. Recent progress in genetics allowed establishment of gene knockdown by RNA interference (RNAi) (Takahashi et al., 2009; Awata et al., 2016) and genome editing by the CRISPR/cas9 system (Awata et al., 2015) in crickets, adding to the well-established pharmacological methods (Unoki et al., 2005, 2006; Matsumoto et al., 2006, 2009, 2016; Mizunami et al., 2014; Sugimachi et al., 2016). Third, there has been a good accumulation of knowledge that bridges between the nervous system and behavior of crickets gained by extensive neuroethological studies in crickets (Stevenson and Schildberger, 2013; Hedwig, 2016).

### Experimental Procedures

In our previous works in crickets, we have developed and extensively studied the olfactory associative conditioning, in which an odor is paired with reinforcement stimulus (Matsumoto and Mizunami, 2000, 2002b; Matsumoto et al., 2015). Similar conditioning protocols applied to two different types of visual stimuli, visual-pattern (Unoki et al., 2006) or color-vision (Nakatani et al., 2009), paired with reinforcement stimuli have also been established. All of these procedures use classical conditioning for training and operant testing for memory tests (Matsumoto and Mizunami, 2002b; Matsumoto et al., 2003) and is performed on individual, isolated cricket. This protocol is built on the fact that crickets are able to transfer memory formed by classical conditioning in a beaker, half-compelled to receive the training, to the environment that allows freedom of choice in a larger testing chamber.

We will slightly go through the details of conditioning taking olfactory appetitive conditioning of an odor with water reward as an example. Before the experiment, crickets are each isolated in a beaker without water for 3 days, which enhances water consumption. A syringe containing water with a piece of filter paper set near the needle tip is used in conditioning training. Odor essence is applied to the filter paper to present the odor. The cricket receives the odor around its antennae for 3 s, and then receives a drop of water reward to the mouth. On water application, crickets attempt to drink it indicating that water serves as an appetitive stimulus. Retention scores of memory formed by single pairing of an odor with water reward (single-trial conditioning) is as high as that formed by repeated pairings of odor-reward association (multiple-trial conditioning) at 30 min after training, but it declines over a period of several hours and is no longer observed at 1 day after training (Matsumoto et al., 2006).

Multiple-trial conditioning consist of two or more repetition of odor-reinforcement trials with inter-trial intervals (ITIs) that induces long-lasting memory beyond 1 day under adequate conditions (e.g., number of trials = 4, ITI = 5 min). Multiple-trial conditioning in our previous studies includes absolute conditioning (A+) and differential conditioning (A+, B-). Absolute conditioning can be described as repetition of appetitive conditioning trials. Differential conditioning combines appetitive and aversive conditioning trials in an alternating order. For olfactory aversive conditioning of an odor with sodium chloride punishment, similar syringe containing 20% sodium chloride solution is used. The crickets show immediate retraction from sodium chloride solution, indicating that it functions as an aversive stimulus. In previous works, we used differential conditioning that leads to robust memory (Matsumoto et al., 2006), but we eventually switched to absolute conditioning for the simplicity of analysis (Matsumoto et al., 2009; Mizunami et al., 2014).

Before and after olfactory associative conditioning, crickets were tested for their odor preferences between two odors during a 4 min testing period. Tests were performed operantly, allowing a cricket to search and choose from two odor sources, a control odor and a conditioned odor, provided in the testing chamber. Relative odor preference index for each cricket was calculated from the visiting time for each of the odor sources, as a ratio of rewarded-odor visiting time to the total visiting time. Visiting time was recorded when odor source was explored by the mouth parts of the cricket.

In our pharmacological behavioral experiments, basically, we injected 3 μl of saline containing a drug into the hemolymph of the cricket’s head using a microsyringe 20 min before the onset of training (see **Table 1** legend for drug doses). All of the drugs used in our experiments had been confirmed for their efficacy in physiological or biochemical researches in insects.

### Memory Phases

As is the case with other animals (DeZazzo and Tully, 1995), memory induced by multiple-trial conditioning in crickets can be further distinguished into several memory phases with different retention curves. In our previous work applying differential conditioning in crickets, we have demonstrated that olfactory memory can be subdivided into at least two memory phases, STM and LTM. The peak memory score induced by sufficient multiple-trial conditioning with sufficient ITIs is retained without decline for a few days (Matsumoto and Mizunami, 2002b), but when injected with a protein synthesis inhibitor (e.g., cycloheximide), memory retention score started to diminish from 5 h after training, and completely disappeared at 8 h after training (Matsumoto et al., 2003). The results indicate that there are two types of memory phases discriminated by the sensitivity to a protein synthesis inhibitor. One type is named LTM that requires protein synthesis and at least maintained for several days (Matsumoto and Mizunami, 2002b). The other type is STM which does not require novel protein synthesis (Matsumoto et al., 2003). The STM peaks immediately after the training until 4 h after training and disappears at 8 h after training. Differential conditioning may be a rather complicated learning task involving both appetitive and aversive learning. Thus, we are switching the conditioning paradigm to the simpler absolute conditioning in recent works. The memory phases in absolute conditioning should be clarified by further investigation.

### cAMP Signaling Pathway

The cAMP signaling system has been demonstrated to be essential in LTM formation in mice (Abel et al., 1997), *Drosophila* (Yin et al., 1995; Isabel et al., 2004) and *Aplysia* (Bartsch et al., 1995). LTM formation in all of these species requires phosphorylation of transcription factor CREB (cAMP-responsive element-binding protein) by PKA (cAMP-dependent protein kinase) which is activated by an increase of intracellular cAMP (Bartsch et al., 1995; Yin et al., 1995; Abel et al., 1997).

**FIGURE 1:**
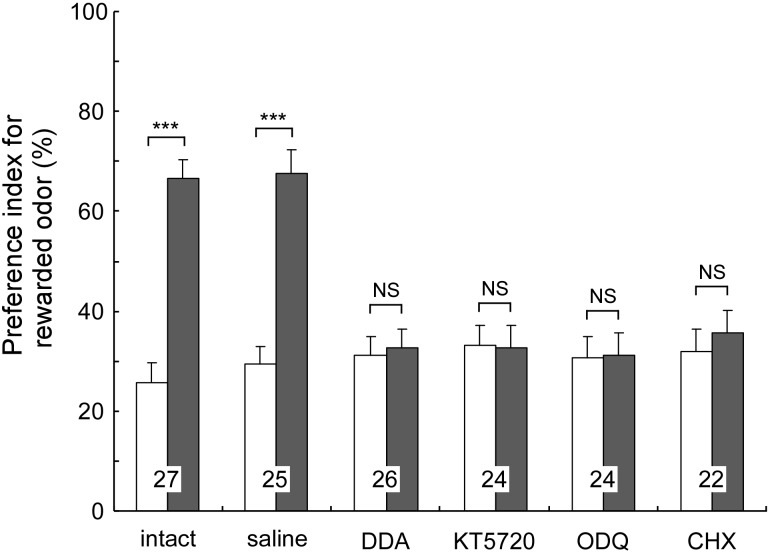
Effects of drug injection prior to multiple-trial conditioning on LTM. Injected drugs are the cAMP signaling inhibitors (DDA, KT5720), the cGMP signaling inhibitor (ODQ) and the protein synthesis inhibitor (CHX). Odor preferences of crickets were tested before conditioning (white bars) and at 1-day after conditioning (shaded bars). The results of statistical comparisons are shown as means + SE. Significant differences of the PIs are indicated by asterisks (WCX test). ^NS^*p* > 0.05, ^∗∗∗^*p* < 0.001. The number of animals is shown at each data point. Modified from Matsumoto et al. (2009).

We investigated whether cAMP signaling is necessary for LTM formation in the cricket (Matsumoto et al., 2006, 2009). Crickets were each injected with inhibitors of key enzymes of cAMP signaling into the hemolymph prior to multiple-trial conditioning. We used either 2′,5′-dideoxyadenosine (DDA) or SQ22536 as an adenylyl cyclase (AC) inhibitor, and either KT5720 or Rp-8-br-cAMPS as a PKA inhibitor. In a retention test 1 day after training, all of the groups of crickets failed to exhibit increased preference to the conditioned odor in comparison to that before conditioning (**Figure 1**). On the other hand, they showed normal scores of 30-min memory retention similar to the control group that had received injection of cricket saline. These observations indicate that these drugs fully impair LTM formation but have no effect on STM formation, motivation, sensory or motor functions. On the other hand, when these drugs were administered after conditioning, they did not impair LTM, indicating that it is during conditioning that activation of cAMP signaling is necessary for LTM formation.

The results of our experiments using ‘LTM-inhibiting’ drugs showed that cAMP signaling is necessary for LTM formation in the cricket, but is it also sufficient for LTM formation? To address this issue, we investigated whether forced LTM formation occurs by upregulating the cAMP signaling pathway during single-trial conditioning, which does not form LTM. Crickets were each injected with an AC activator (forskolin) or a cAMP analog (DB-cAMP, 8-br-cAMP) into the hemolymph prior to single-trial conditioning. In a retention test 1 day after the conditioning, higher preference scores for the conditioned odor in comparison to that before conditioning were observed in all of the groups, and their scores were as high as that of crickets that had been trained by multiple-trial conditioning (Matsumoto et al., 2006; Mizunami et al., 2014). Moreover, crickets co-injected with a protein synthesis inhibitor (cyclohexymide) and one of the activators of cAMP signaling paired with single-trial conditioning did not exhibit 1-day memory retention. These results suggest that activators of cAMP signaling induce protein-dependent LTM.

### NO-cGMP Signaling Pathway

NO-cGMP signaling is also critical for producing LTM in sheep (Kendrick et al., 1997), *Lymnaea* (Kemenes et al., 2002) and honey bees (Müller, 1996, 2000). NO is both intra- and intercellular signaling molecule with high reactivity and membrane-permeable property, synthesized by NO synthase (NOS). Through paracrine effect of NO, soluble guanylyl cyclase (sGC) in adjacent cells produce cGMP which is involved in various physiological functions (Garthwaite et al., 1988; Garthwaite and Boulton, 1995), including induction of LTM in many animals (Bernabeu et al., 1996; Prickaerts et al., 2002).

To investigate whether NO-cGMP signaling is necessary for LTM formation in the cricket, crickets were each injected with an NOS inhibitor (L-NAME) or an sGC inhibitor (ODQ) prior to multiple-trial conditioning (Matsumoto et al., 2006, 2009). These groups of crickets did not show 1-day memory retention, whereas 30-min memory retention remained intact (Matsumoto et al., 2006, 2009). These observations indicate that inhibition of NO-cGMP signaling fully impairs LTM formation but has no effect on STM formation. We also obtained comparable results using RNAi: injection of NOS dsRNA fully impaired 1-day retention but not 30-min retention in 7th-instar nymphal crickets (Takahashi et al., 2009).

The results of our experiments using ‘LTM-inhibiting’ drugs showed that NO-cGMP signaling is required to establish LTM in the cricket. Next, we investigated whether externally applied activators of NO-cGMP signaling paired with single-trial conditioning can facilitate LTM formation. Crickets each injected with an NO donor (SNAP, NOR3) or a cGMP analog (8-br-cGMP) before the single-trial conditioning showed significantly high retention level at 1 day after conditioning, which was almost identical to that in saline-injected group at 1 day after multiple-trial conditioning (Matsumoto et al., 2006). Moreover, crickets co-injected with a protein synthesis inhibitor (cyclohexymide) and an activator of NO-cGMP signaling paired with single-trial conditioning did not exhibit 1-day memory retention, indicating that activators of NO-cGMP signaling pathway induce formation of protein-dependent memory, that is, LTM.

### NO-cGMP Signaling Stimulates cAMP Signaling to Induce LTM

Our pharmacological behavioral experiments using ‘LTM-inhibiting’ drugs or ‘LTM-inducing’ drugs suggested that NO-cGMP signaling and cAMP signaling are both necessary and sufficient for cricket LTM formation, particularly in the conditioning process.

Next, to determine which of the two pathways, NO-cGMP signaling or cAMP signaling, precedes the other in the LTM formation cascade, we varied the combinations of ‘LTM-inhibiting’ drugs or ‘LTM-inducing’ drugs paired with single-trial conditioning and evaluated their effects. For example, we investigated whether cAMP mediates the forced LTM formation by combining a cGMP analog injection with single-trial conditioning (Matsumoto et al., 2006). While LTM induction by combination of a cGMP analog (8-br-cGMP) and single-trial conditioning was unaffected by co-injection of an NOS inhibitor (L-NAME), it was completely impaired by co-injection of an AC inhibitor (DDA).

Induction of LTM by single-trial conditioning paired with ‘LTM-inducing’ drugs related to cAMP signaling (AC activator forskolin, cAMP analog DB-cAMP) was unaffected by ‘LTM-inhibiting’ drugs related to NO-cGMP signaling (L-NAME, ODQ) (Matsumoto et al., 2006). In contrast, induction of LTM by single-trial conditioning paired with ‘LTM-inducing’ drugs related to NO-cGMP signaling (SNAP, 8-br-cGMP) was fully blocked by ‘LTM-inhibiting’ drugs related to cAMP signaling (DDA, KT5720). The results suggest that in the LTM induction process, the AC-cAMP pathway works downstream of the NO-cGMP pathway, and not vice versa.

### Biological Pathways Intervening Between NO-cGMP Signaling and cAMP Signaling

Next, we investigated biological pathways intervening between cGMP and AC activation. PKG, a cGMP-dependent protein kinase, is one of the possible targets of cGMP. Working in parallel with PKA, PKG enhances the phosphorylation of CREB in mice (Lu and Hawkins, 2002). Working in parallel with the cAMP pathway, NO-cGMP-PKG signaling pathway governs the induction of long-term hyper-excitability on receiving a noxious stimulation in nociceptive sensory neurons of *Aplysia* (Lewin and Walters, 1999). We investigated the roles of PKG in olfactory memory in the cricket. LTM formation was not affected by external application of PKG inhibitor KT5823, whether it was induced by multiple-trial conditioning or by single-trial conditioning combined with 8-br-cGMP.

Thus, we switched our target to cyclic nucleotide-gated cation channel (CNG channel). CNG channels are Ca^2+^-permeable channels activated by cAMP and/or cGMP. A CNG channel inhibitor [L*-cis* diltiazem (L-DIL), 3,4,-dechlorobenzamil (DCB)] fully impaired LTM, but not STM, formed by multiple-trial conditioning. Moreover, the CNG channel inhibitor L-DIL fully impaired LTM induced by combination of a cGMP analog (8-br-cGMP) and single-trial conditioning, while L-DIL did not affect LTM induced by ‘LTM-inducing’ drugs related to cAMP signaling (forskolin, DB-cAMP) paired with single-trial conditioning. From the results, it can be suggested that CNG channel plays its role downstream of cGMP and upstream of AC activation in the LTM formation process.

In *Drosophila*, it has been shown that AC is activated by either G-protein or calcium-calmodulin (Ca^2+^/CaM) (Livingstone et al., 1984). CaM is a principal Ca^2+^-binding messenger protein in the central nervous system. We examined whether CaM mediates the signaling pathway from CNG channel to AC activation. A CaM inhibitor (W-7) fully impaired LTM formed by multiple-trial conditioning. Moreover, the CaM inhibitor W-7 fully impaired LTM induced by a cGMP analog (8-br-cGMP) paired with single-trial conditioning, while it had no effect on LTM induced by ‘LTM-inducing’ drugs related to cAMP signaling (forskolin, DB-cAMP) paired with single-trial conditioning. Next, we investigated whether rise in calcium concentration mediates signaling from CNG channel to CaM in LTM formation process. Crickets injected with a calcium (Ca^2+^) ionophore (A23187) paired with single-trial conditioning exhibited LTM. The LTM induced by A23187 was unaffected by co-injection of an sGC inhibitor (ODQ) or a CNG channel inhibitor (L-DIL) but was completely impaired by co-injection of a CaM inhibitor (W-7) or an AC inhibitor (DDA). The results indicate that Ca^2+^/CaM mediates signaling from CNG channel to AC, filling the gap of LTM formation cascade.

Ca^2+^/CaM-dependent serine/threonine kinase II (CaMKII), which is one of the Ca^2+^/CaM effector enzymes, supports various learning and memory systems as a key signaling molecule in vertebrates (Coultrap and Bayer, 2012). This is especially because CaMKII have the ability to modulate its own kinase activity by autophosphorylation. In the fruit fly *Drosophila*, synthesis of CaMKII in mushroom bodies has been reported to be necessary for olfactory LTM formation (Ashraf et al., 2006; Akalal et al., 2010; Malik et al., 2013). The mushroom body is known as a multisensory association center as well as a secondary olfactory center essential for olfactory learning and memory (Heisenberg, 2003; Davis, 2011). In cockroaches, an increase of phosphorylated CaMKII is observed in pre- and post-synaptic structures in the mushroom body calyx after learning to associate an olfactory stimulus with a visual stimulus (Lent et al., 2007). In our recent report, we demonstrated that CaMKII inhibitors impair the olfactory LTM formation in honey bees (Matsumoto et al., 2014). Are these roles of CaMKII in olfactory memory processing introduced above also true for crickets? In crickets, a CaMKII inhibitor (KN-62 or KN-93) fully impaired induction of LTM, but not STM, paired with multiple-trial conditioning. Moreover, KN-62 fully impaired induction of LTM by a Ca^2+^ ionophore (A23187) paired with single-trial conditioning, but not that by a cAMP analog, indicating that CaMKII works upstream of AC for LTM formation cascade. Because KN-62 did not impair LTM induced by a cAMP analog, it was rather surprising to find out that KN-62 or KN-93 inhibits LTM induction with folskolin, an AC activator. The best working theory to explain these observations is that there is an interaction between CaMKII and AC, conceivably through formation of macromolecular complex in a similar manner demonstrated in mammalian CaMKII (Coultrap and Bayer, 2012; Lisman et al., 2012), and when KN-62 or KN-93 binds to CaMKII, AC activation by forskolin may be impaired.

### A Model of the Signaling Pathways for LTM Formation

A putative model of the signaling pathways for olfactory LTM formation in crickets is shown in **Figure 1**, updated from our previous model (Mizunami et al., 2014). The new model illustrates the simplest of all the signaling pathways that account for the results summarized in **Table 1**, which describes the outcomes of co-injection experiments. The following documented findings in several insects are incorporated in this model: (1) *in vitro* alpha-bungarotoxin (BGT)-sensitive nicotinic acetylcholine receptors (nAChRs) are able to trigger NO synthesis in Kenyon cells of insects (Bicker et al., 1996; Zayas et al., 2002), (2) NO production by NO synthase is stimulated by Ca^2+^/CaM in *Drosophila* (Regulski and Tully, 1995), (3) *in vitro* muscarinic acetylcholine receptors (mAChR) activate CaM by calcium release from the endoplasmic reticulum (ER) via PLC/IP_3_ signaling (Hasebe and Yoshino, 2016), (4) calcium release via ryanodine receptors (RyRs) on the ER induces LTM in crickets (Sugimachi et al., 2016), (5) AC is activated by either the G-protein coupled receptor or Ca^2+^/CaM in *Drosophila* (Livingstone et al., 1984) and (6) PKA activates CREB which leads to LTM formation in *Drosophila* (Yin et al., 1995).

Anatomical studies of NO-generating neurons and NO-receptive neurons have been performed in some insects. Putative NO synthase have been revealed histochemically in some neurons of the mushroom body and the antennal lobe, a primary olfactory center, in honey bees (Bicker, 2001), locusts (Müller and Bicker, 1994) and cockroaches (Ott and Elphick, 2002), while immunoreactivity to NO-induced cGMP has been observed in other neurons of the same centers (Bicker et al., 1996; Bicker, 2001). To determine the brain region of NO-generating neurons and NO-receptive neurons in crickets, we investigated the expression patterns of the *NOS* gene and *SGCβ* gene by whole-mount *in situ* hybridization (Takahashi et al., 2009). The *SGCβ* gene is coding the β subunit of sGC. We observed a high expression level of *NOS* mRNA in outer Keyon cells of the mushroom body, but not in inner Kenyon cells, in addition to several somata around the antennal lobe and at the base of the visual center optic lobe. On the other hand, we observed a significant level of expression of *sGC* mRNA in inner Keyon cells. Therefore, NO production is presumed to take place in outer Kenyon cells, and NO permeates into nearby inner Kenyon cells.

**FIGURE 2:**
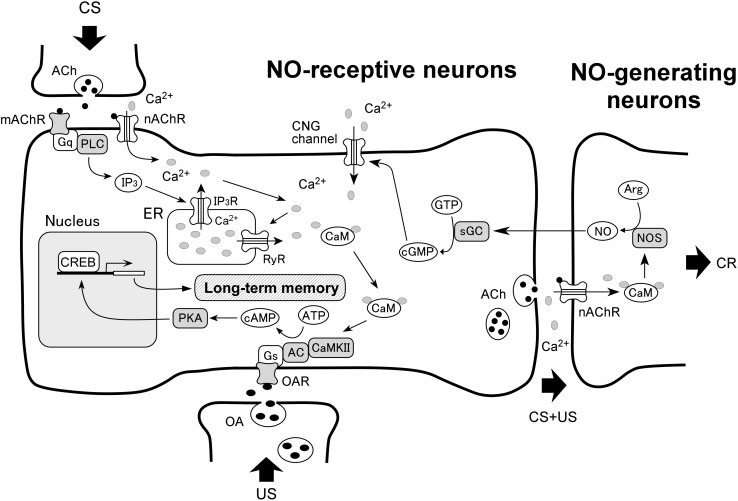
A model of biochemical pathways for LTM formation in associative olfactory conditioning. The model is proposed on the basis of the present findings in crickets and some documented findings in insects (see text). Single-trial conditioning induces only short-term synaptic plasticity that underlies protein synthesis-independent short-term memory (STM). Multiple-trial conditioning activates NO-cGMP signaling, and this activates cyclic nucleotide-gated (CNG) channel, Ca^2+^/CaM, CaMKII and then adenylyl cyclase (AC)-cAMP-PKA signaling. This in turn activates cAMP-responsive element-binding protein (CREB), which results in transcription and translation of genes that are necessary for achieving long-term plasticity of synaptic connection upon other neurons that underlies LTM. NOS, NO synthase; sGC, soluble guanylyl cyclase; Arg, arginine; Gs, Gq, receptor (R)-coupled G-protein; OA, octopamine; ACh, acetylcholine; nAChR, nicotinic acetylcholine receptor; mAChR, muscarinic acetylcholine receptor; PLC, phospholipase C; IP3, inositol 1,4,5-triphosphate; RyR, ryanodine receptor; ER, endoplasmic reticulum.

One of our next steps is to clarify whether several biological molecules depicted in **Figure 2** indeed contribute to LTM formation in crickets using both pharmacological study and RNAi. The target molecules include nAChR, mAChR, PLC, IP_3_ and CREB, which have not been shown to be involved in cricket LTM formation. There are several LTM-related signaling pathways other than those mentioned in this review in other animals, such as *N*-methyl-D-aspartic acid (NMDA) receptor signaling (Giese et al., 2015; Wang and Peng, 2016), insulin receptor signaling (Zhao and Alkon, 2001; Zhao et al., 2004; Dou et al., 2005; Chambers et al., 2015; Kojoma et al., 2015), mitogen-activated protein kinase (MAPK) signaling (Alfieri et al., 2011; Philips et al., 2013; Shobe et al., 2016), and mechanistic target of rapamycin (mTOR) signaling (Bekinschtein et al., 2007; Blundell et al., 2008; Huang et al., 2013; Buffington et al., 2014; Hylin et al., 2018). Whether these signaling pathways are related to LTM formation in crickets is another issue.

We have established conditioning procedures for different sensory modalities for crickets: olfactory conditioning, visual-pattern conditioning and color-vision conditioning. Each conditioning can be classified into two categories: appetitive conditioning and aversive conditioning. Thus, we can examine whether the finding of biochemical cascades in olfactory appetitive learning is applicable to other learning paradigms. For example, in appetitive visual LTM formation, we have shown that NO-cGMP signaling works upstream of cAMP signaling (Matsumoto et al., 2013b). We have also shown that at least NO-cGMP signaling participates in aversive visual LTM formation (Matsumoto et al., 2013b). Thus, we conclude that signaling cascades for LTM formation is shared between olfactory and visual learning.

## Conclusion

In this review, we overviewed the biochemical cascades for LTM formation based on the results of co-injection experiments with different combinations of LTM-inducing drugs for ‘gain of function’ and LTM-inhibiting drugs for ‘loss of function.’ From our pharmacological behavioral studies, we proposed an updated model in which multiple-trial conditioning triggers the NO-cGMP signaling that activates the downstream cAMP signaling through the CNG channel, Ca^2+^/CaM and CaMKII, leading to the formation of protein synthesis-dependent LTM. A number of molecular actors involved in LTM formation in crickets, such as NOS, NO, cGMP, cAMP, PKA and CaMKII, are known to be involved in mammalian LTM formation. Thus, we conclude that insects, with relatively simple brain structures and neural circuitry, will also be beneficial in exploratory experiments to predict the molecular mechanisms underlying cognitive functions and memory formation in mammals.

## Author Contributions

YM, CM, and MM wrote the manuscript and approved the final version.

## Conflict of Interest Statement

The authors declare that the research was conducted in the absence of any commercial or financial relationships that could be construed as a potential conflict of interest. The reviewer MV and handling Editor declared their shared affiliation.

## References

[B1] Abel T. Nguyen P. V. Barad M. Deuel T. A. Kandel E. R. Bourtchoudze R. (1997). Genetic demonstration of a role for PKA in the late phase of LTP and in hippocampus-based long-term memory. *Cell* 88 615–626. 10.1016/S0092-8674(00)81904-2 9054501

[B2] Akalal D. B. Yu D. Davis R. L. (2010). A late-phase, long-term memory trace forms in the γ neurons of *Drosophila* mushroom bodies after olfactory classical conditioning. *J. Neurosci.* 30 16699–16708. 10.1523/JNEUROSCI.1882-10.2010 21148009 PMC3380342

[B3] Alfieri P. Cesarini L. Mallardi M. Piccini G. Caciolo C. Leoni C. (2011). Long term memory profile of disorders associated with dysregulation of the RAS-MAPK signaling cascade. *Behav. Genet.* 41 423–429. 10.1007/s10519-011-9446-5 21274610

[B4] Ashraf S. I. McLoon A. L. Sclarsic S. M. Kunes S. (2006). Synaptic protein synthesis associated with memory is regulated by the RISC pathway in *Drosophila*. *Cell* 124 191–205. 10.1016/j.cell.2005.12.017 16413491

[B5] Awata H. Wakuda R. Ishimaru Y. Matsuoka Y. Terao K. Katata S. (2016). Roles of OA1 octopamine receptor and Dop1 dopamine receptor in mediating appetitive and aversive reinforcement revealed by RNAi studies. *Sci. Rep.* 6:29696. 10.1038/srep29696 27412401 PMC4944188

[B6] Awata H. Watanabe T. Hamanaka Y. Mito T. Noji S. Mizunami M. (2015). Knockout crickets for the study of learning and memory: Dopamine receptor Dop1 mediates aversive but not appetitive reinforcement in crickets. *Sci. Rep.* 5:15885. 10.1038/srep15885 26521965 PMC4629116

[B7] Bartsch D. Gjorardo M. Skehel P. A. Karl K. A. Herder S. P. Chen M. (1995). Aplysia CREB2 represses long-term facilitation: relief of repression converts transient facilitation into long-term functional and structural change. *Cell* 83 979–992. 10.1016/0092-8674(95)90213-9 8521521

[B8] Bekinschtein P. Katche C. Slipczuk L. N. Igaz L. M. Cammarota M. Izquierdo I. (2007). mTOR signaling in the hippocampus is necessary for memory formation. *Neurobiol. Learn. Mem.* 87 303–307. 10.1016/j.nlm.2006.08.007 17005423

[B9] Bernabeu R. Schmitz P. Faillace M. P. Izquierdo I. Medina J. H. (1996). Hippocampal cGMP and cAMP are differentially involved in memory processing of inhibitory avoidance learning. *Neuroreport* 7 585–588. 10.1097/00001756-199601310-00050 8730835

[B10] Bicker G. (2001). Sources and targets of nitric oxide signaling in insect nervous systems. *Cell Tissue Res.* 303 137–146. 10.1007/s004410000321 11291761

[B11] Bicker G. Schmachtenberg O. DeVerte J. (1996). The nitric oxide/cyclic GMP messenger system in olfactory pathway of the locust brain. *Eur. J. Neurosci.* 8 2635–2643. 10.1111/j.1460-9568.1996.tb01558.x 8996813

[B12] Blundell J. Kouser M. Powell C. M. (2008). Systemic inhibition of mammalian target of rapamycin inhibits fear memory reconsolidation. *Neurobiol. Learn. Mem.* 90 28–35. 10.1016/j.nlm.2007.12.004 18316213 PMC2497420

[B13] Buffington S. A. Huang W. Costa-Mattioli M. (2014). Translational control in synaptic plasticity and cognitive dysfunction. *Annu. Rev. Neurosci.* 37 17–38. 10.1146/annurev-neuro-071013-014100 25032491 PMC4721605

[B14] Chambers D. B. Androschuk A. Rosenfelt C. Langer S. Harding M. Bolduc F. V. (2015). Insulin signaling is acutely required for long-term memory in *Drosophila*. *Front. Neural Circuits* 9:8. 10.3389/fncir.2015.00008 25805973 PMC4354381

[B15] Coultrap S. J. Bayer K. U. (2012). CaMKII regulation in information processing and storage. *Trends Neurosci.* 35 607–618. 10.1016/j.tins.2012.05.003 22717267 PMC3461103

[B16] Davis R. L. (2011). Traces of *Drosophila* memory. *Neuron* 70 8–19. 10.1016/j.neuron.2011.03.012 21482352 PMC3374581

[B17] DeZazzo J. Tully T. (1995). Dissection of memory formation: from behavioral pharmacology to molecular genetics. *Trends Neurosci.* 18 212–218. 10.1016/0166-2236(95)93905-D 7610491

[B18] Dou J. T. Chen M. Dufour F. Alkon D. L. Zhao W. Q. (2005). Insulin receptor signaling in long-term memory consolidation following spatial learning. *Learn. Mem.* 12 646–655. 10.1101/lm.88005 16287721 PMC1356184

[B19] Garthwaite J. Boulton C. L. (1995). Nitric oxide signaling in the central nervous system. *Annu. Rev. Physiol.* 57 683–706. 10.1146/annurev.ph.57.030195.003343 7539993

[B20] Garthwaite J. Charles S. L. Chess-Williams R. (1988). Endothelium-derived relaxing factor release on activation of NMDA receptors suggests a role as intracellular messenger in the brain. *Nature* 336 385–388. 10.1038/336385a0 2904125

[B21] Giese K. P. Aziz W. Kraev I. Stewart M. G. (2015). Generation of multi-innervated dendritic spines as a novel mechanism of long-term memory formation. *Neurobiol. Learn. Mem.* 124 48–51. 10.1016/j.nlm.2015.04.009 25933505

[B22] Hasebe M. Yoshino M. (2016). Nitric oxide/cGMP/PKG signaling pathway activated by M1-type muscarinic acetylcholine receptor cascade inhibits Na+-activated K+ currents in Kenyon cells. *J. Neurophysiol.* 115 3174–3185. 10.1152/jn.00036.2015 26984419 PMC4946596

[B23] Hedwig B. G. (2016). Sequential filtering processes shape feature detection in crickets: a framework for song pattern recognition. *Front. Physiol.* 7:46. 10.3389/fphys.2016.00046 26941647 PMC4766296

[B24] Heisenberg M. (2003). Mushroom body memoir: from maps to models. *Nat. Rev. Neurosci.* 4 266–275. 10.1038/nrn1074 12671643

[B25] Huang W. Zhu P. J. Zhang S. Zhou H. Stoica L. Galiano M. (2013). mTORC2 controls actin polymerization required for consolidation of long-term memory. *Nat. Neurosci.* 16 441–448. 10.1038/nn.3351 23455608 PMC3615448

[B26] Hylin M. J. Zhao J. Tangavelou K. Rozas N. S. Hood K. N. MacGowan J. S. (2018). A role for autophagy in long-term spatial memory formation in male rodents. *J. Neurosci. Res.* 96 416–426. 10.1002/jnr.24121 29230855 PMC6425965

[B27] Isabel G. Pascual A. Preat T. (2004). Exclusive consolidated memory phases in *Drosophila*. *Science* 304 1024–1027. 10.1126/science.1094932 15143285

[B28] Kandel E. R. (2001). The molecular biology of memory storage; a dialogue between genes and synapses. *Science* 294 1030–1038. 10.1126/science.1067020 11691980

[B29] Kemenes I. Kemenes G. Andrew R. J. Benjamin P. R. O’Shea M. (2002). Critical time-window for NO-cGMP dependent long-term memory formation after one-trial appetitive conditioning. *J. Neurosci.* 22 1414–1425. 10.1523/JNEUROSCI.22-04-01414.2002 11850468 PMC6757551

[B30] Kendrick K. M. Guevara-Guzman R. Zorrilla J. Hinton M. R. Broad K. D. Mimmack M. (1997). Formation of olfactory memories mediated by nitric oxide. *Nature* 388 670–674. 10.1038/41765 9262400

[B31] Kojoma S. Sunada H. Mita K. Sakakibara M. Lukowiak K. Ito E. (2015). Function of insulin in snail brain in associative learning. *J. Comp. Physiol. A* 201 959–981. 10.1007/s00359-015-1032-5 26233474

[B32] Lent D. D. Pintér M. Strausfeld N. J. (2007). Learning with half a brain. *Dev. Neurobiol.* 67 740–751. 10.1002/dneu.20374 17443821

[B33] Lewin M. R. Walters E. (1999). Cyclic GMP pathway is critical for inducing long-term sensitization of nociceptive sensory neurons. *Nat. Neurosci.* 2 18–23. 10.1038/4520 10195175

[B34] Lisman J. Yasuda R. Raghavachari S. (2012). Mechanisms of CaMKII action in long-term potentiation. *Nat. Rev. Neurosci.* 13 169–182. 10.1038/nrn3192 22334212 PMC4050655

[B35] Livingstone M. S. Sziber P. P. Quinn W. G. (1984). Loss of calcium/calmodulin responsiveness in adenylate cyclase of rutabaga, a *Drosophila* learning mutant. *Cell* 37 205–215. 10.1016/0092-8674(84)90316-7 6327051

[B36] Lu Y.-F. Hawkins R. D. (2002). Ryanodine receptors contribute to cGMP-induced late-phase LTP and CREB phosphorylation in the hippocampus. *J. Neurosci.* 88 1270–1278. 10.1152/jn.2002.88.3.1270 12205148

[B37] Malik B. R. Gillespie J. M. Hodge J. J. (2013). CASK and CaMKII function in the mushroom body α’/β’ neurons during *Drosophila* memory formation. *Front. Neural Circuits* 7:52. 10.3389/fncir.2013.00052 23543616 PMC3608901

[B38] Matsumoto Y. Hatano A. Unoki S. Mizunami M. (2009). Stimulation of the cAMP system by the nitric oxide-cGMP system underlying the formation of long-term memory in an insect. *Neurosci. Lett.* 467 81–85. 10.1016/j.neulet.2009.10.008 19818830

[B39] Matsumoto Y. Hirashima D. Mizunami M. (2013a). Analysis and modeling of neural processes underlying sensory preconditioning. *Neurobiol. Learn. Mem.* 101 103–113. 10.1016/j.nlm.2013.01.008 23380289

[B40] Matsumoto Y. Hirashima D. Terao K. Mizunami M. (2013b). Roles of NO signaling in long-term memory formation in visual learning in an insect. *PLoS One* 8:e68538. 10.1371/journal.pone.0068538 23894314 PMC3722230

[B41] Matsumoto Y. Matsumoto C. S. Takahashi T. Mizunami M. (2016). Activation of NO-cGMP signaling rescues age-related memory impairment in an insect. *Front. Behav. Neurosci.* 10:166. 10.3389/fnbeh.2016.00166 27616985 PMC4999442

[B42] Matsumoto Y. Matsumoto C. S. Wakuda R. Ichihara S. Mizunami M. (2015). Roles of octopamine and dopamine in appetitive and aversive memory acquisition studied in olfactory conditioning of maxillary palpi extension response in crickets. *Front. Behav. Neurosci.* 9:230. 10.3389/fnbeh.2015.00230 26388749 PMC4555048

[B43] Matsumoto Y. Mizunami M. (2000). Olfactory learning in the cricket *Gryllus bimaculatus*. *J. Exp. Biol.* 203 2581–2588. 10934001 10.1242/jeb.203.17.2581

[B44] Matsumoto Y. Mizunami M. (2002a). Lifetime olfactory memory in the cricket *Gryllus bimaculatus*. *J. Comp. Physiol. A* 188 295–299. 10.1007/s00359-002-0303-0 12012100

[B45] Matsumoto Y. Mizunami M. (2002b). Temporal determinants of olfactory long-term retention in the cricket *Gryllus bimaculatus*. *J. Exp. Biol.* 205 1429–1437. 11976354 10.1242/jeb.205.10.1429

[B46] Matsumoto Y. Mizunami M. (2004). Context-dependent olfactory learning in an insect. *Learn. Mem.* 11 288–293. 10.1101/lm.72504 15169858 PMC419731

[B47] Matsumoto Y. Mizunami M. (2006). Olfactory memory capacity of the cricket *Gryllus bimaculatus*. *Biol. Lett.* 2 608–610. 10.1098/rsbl.2006.0540 17148301 PMC1834001

[B48] Matsumoto Y. Noji S. Mizunami M. (2003). Time course of protein synthesis-dependent phase of olfactory memory in the cricket *Gryllus bimaculatus*. *Zool. Sci.* 20 409–416. 10.2108/zsj.20.409 12719642

[B49] Matsumoto Y. Sandoz J.-C. Devaud J.-M. Lormant F. Mizunami M. Giurfa M. (2014). Cyclic nucleotide-gated channels, calmodulin, adenylyl cyclase, and calcium/calmodulin-dependent protein kinase II are required for late, but not early, long-term memory formation in the honeybee. *Learn. Mem.* 21 272–284. 10.1101/lm.032037.113 24741108 PMC3994501

[B50] Matsumoto Y. Unoki S. Aonuma H. Mizunami M. (2006). Critical roles of the nitric oxide-cGMP cascade in the formation of cAMP-dependent long-term memory. *Learn. Mem.* 13 35–44. 10.1101/lm.130506 16452652 PMC1360131

[B51] Mizunami M. Nemoto Y. Terao K. Hamanaka Y. Matsumoto Y. (2014). Roles of calcium/calmodulin-dependent kinase II in long-term memory formation in crickets. *PLoS One* 9:9. 10.1371/journal.pone.0107442 25215889 PMC4162583

[B52] Mizunami M. Unoki S. Mori Y. Hirashima D. Hatano A. Matsumoto Y. (2009). Roles of octopaminergic and dopaminergic neurons in appetitive and aversive memory recall in an insect. *BMC Biol.* 7:46. 10.1186/1741-7007-7-46 19653886 PMC2729297

[B53] Montarolo P. G. Goelet P. Casterllucci V. F. Morgan J. Kandel E. R. Schacher S. (1986). A critical period for macromolecular synthesis in long-term heterosynaptic facilitation in Aplysia. *Science* 234 1249–1254. 10.1126/science.3775383 3775383

[B54] Müller U. (1996). Inhibition of nitric oxide synthase impairs a distinct form of long-term memory in the honeybee *Apis mellifera*. *Neuron* 16 541–549. 10.1016/S0896-6273(00)80073-2 8785051

[B55] Müller U. (2000). Prolonged activation of cAMP-dependent protein kinase during conditioning induces long-term memory in honeybees. *Neuron* 27 159–168. 10.1016/S0896-6273(00)00017-9 10939339

[B56] Müller U. Bicker G. (1994). Calcium activated release of nitric oxide and cellular distribution of nitric oxide synthesizing neurons in the nervous system of the locust. *J. Neurosci.* 14 7521–7528. 10.1523/JNEUROSCI.14-12-07521.1994 7527844 PMC6576908

[B57] Nakatani Y. Matsumoto Y. Mori Y. Hirashima D. Nishino H. Arikawa K. (2009). Why the carrot is more effective than the stick: different dynamics of punishment memory and reward memory and its possible biological basis. *Neurobiol. Learn. Mem.* 92 370–380. 10.1016/j.nlm.2009.05.003 19435611

[B58] Ott S. R. Elphick M. R. (2002). Nitric oxide synthase histochemistry in insect nervous systems: methanol/formalin fixation reveals the neuroarchitecture of formaldehyde-sensitive NADPH diaphorase in the cockroach Periplaneta americana. *J. Comp. Neurol.* 448 165–185. 10.1002/cne.10235 12012428

[B59] Philips G. T. Ye X. Kopec A. M. Carew T. J. (2013). MAPK establishes a molecular context that defines effective training patterns for long-term memory formation. *J. Neurosci.* 33 7565–7573. 10.1523/JNEUROSCI.5561-12.2013 23616561 PMC3865502

[B60] Prickaerts J. de Vente J. Honig W. Steinbusch H. W. Blokland A. (2002). cGMP, but not cAMP, in rat hippocampus is involved in early stages of object memory consolidation. *Eur. J. Pharmacol.* 436 83–87. 10.1016/S0014-2999(01)01614-4 11834250

[B61] Regulski M. Tully T. (1995). Molecular and biochemical characterization of dNOS: a *Drosophila* Ca2+/calmodulin-dependent nitric oxide synthase. *Proc. Natl. Acad. Sci. U.S.A.* 92 9072–9076. 10.1073/pnas.92.20.9072 7568075 PMC40926

[B62] Shobe J. Philips G. T. Carew T. J. (2016). Transforming growth factor β recruits persistent MAPK signaling to regulate long-term memory consolidation in *Aplysia californica*. *Learn. Mem.* 23 182–188. 10.1101/lm.040915.115 27084925 PMC4836639

[B63] Stevenson P. A. Schildberger K. (2013). Mechanisms of experience dependent control of aggression in crickets. *Curr. Opin. Neurobiol.* 23 318–323. 10.1016/j.conb.2013.03.002 23537901

[B64] Sugimachi S. Matsumoto Y. Mizunami M. Okada J. (2016). Effects of caffeine on olfactory learning in crickets. *Zoolog. Sci.* 33 513–519. 10.2108/zs150209 27715426

[B65] Takahashi T. Hamada A. Miyawaki K. Matsumoto Y. Mito T. Noji S. (2009). Systemic RNA interference for the study of learning and memory in an insect. *J. Neurosci. Methods* 179 9–15. 10.1016/j.jneumeth.2009.01.002 19437615

[B66] Unoki S. Matsumoto Y. Mizunami M. (2005). Participation of octopaminergic reward system and dopaminergic punishment system in insect olfactory learning revealed by pharmacological study. *Eur. J. Neurosci.* 22 1409–1416. 10.1111/j.1460-9568.2005.04318.x 16190895

[B67] Unoki S. Matsumoto Y. Mizunami M. (2006). Roles of octopaminergic and dopaminergic neurons in mediating reward and punishment signals in insect visual learning. *Eur. J. Neurosci.* 24 2031–2038. 10.1111/j.1460-9568.2006.05099.x 17067299

[B68] Wang H. Peng R. Y. (2016). Basic roles of key molecules connected with NMDAR signaling pathway on regulating learning and memory and synaptic plasticity. *Mil. Med. Res.* 3:26. 10.1186/s40779-016-0095-0 27583167 PMC5006437

[B69] Yin J. C. Del Vecchio M. Zhou H. Tully T. (1995). CREB as a memory modulator: induced expression of a dCREB2 activator isoform enhances long-term memory in *Drosophila*. *Cell* 81 107–115. 10.1016/0092-8674(95)90375-5 7720066

[B70] Zayas R. M. Qazi S. Morton D. B. Trimmer B. A. (2002). Nicotinic-acetylcholine receptors are functionally coupled to the nitric oxide/cGMP-pathway in insect neurons. *J. Neurochem.* 83 421–431. 10.1046/j.1471-4159.2002.01147.x 12423252

[B71] Zhao W. Q. Alkon D. L. (2001). Role of insulin and insulin receptor in learning and memory. *Mol. Cell. Endocrinol.* 177 125–134. 10.1016/S0303-7207(01)00455-5 11377828

[B72] Zhao W. Q. Chen H. Quon M. J. Alkon D. L. (2004). Insulin and the insulin receptor in experimental models of learning and memory. *Eur. J. Pharmacol.* 490 71–81. 10.1016/j.ejphar.2004.02.045 15094074

